# Pathophysiology of Non Alcoholic Fatty Liver Disease

**DOI:** 10.3390/ijms17122082

**Published:** 2016-12-11

**Authors:** Salvatore Petta, Amalia Gastaldelli, Eleni Rebelos, Elisabetta Bugianesi, Piergiorgio Messa, Luca Miele, Gianluca Svegliati-Baroni, Luca Valenti, Ferruccio Bonino

**Affiliations:** 1 Gastroenterology, Di.Bi.M.I.S Policlinic Paolo Giaccone Hospital, University of Palermo, PC 90127 Palermo, Italy; petsa@inwind.it; 2 Cardiometabolic Risk Unit—Institute of Clinical Physiology, CNR, PC 56124 Pisa, Italy; amalia@ifc.cnr.it; 3 Department of Clinical and Experimental Medicine, University of Pisa, PC 56122 Pisa, Italy; elenirebelos@gmail.com; 4 Gastroenterology and Hepatology, Department of Medical Sciences, Città della, Salute e della Scienza di Torino Hospital, University of Turin, PC 10122 Turin, Italy; ebugianesi@yahoo.it; 5 Department of Nephrology, Urology and Renal Transplant—Fondazione IRCCS Ca’, Granda, PC 20122 Milano, Italy; pmessa@policlinico.mi.it; 6 Institute of Internal Medicine, Gastroenterology and Liver Diseases Unit, Fondazione Policlinico Gemelli, Catholic University of Rome, PC 00168 Rome, Italy; luca.miele@policlinicogemelli.it; 7 Department of Gastroenterology 1 and Obesity 2, Polytechnic University of Marche, PC 60121 Ancona, Italy; gsvegliati@gmail.com; 8 Metabolic Liver Diseases—Università degli Studi Milano-Fondazione IRCCS Ca’, Granda via F Sforza 35, PC 20122 Milano, Italy; luca.valenti@unimi.it; 9 Institute for Health, PC 53042 Chianciano Terme, Italy

**Keywords:** fatty liver, insulin resistance, free fatty acids, cholesterol, adiponectin, leptin, insulin, glucagon, glucagon-like peptide 1, ghrelin, irisin, selenoprotein P

## Abstract

The physiopathology of fatty liver and metabolic syndrome are influenced by diet, life style and inflammation, which have a major impact on the severity of the clinicopathologic outcome of non-alcoholic fatty liver disease. A short comprehensive review is provided on current knowledge of the pathophysiological interplay among major circulating effectors/mediators of fatty liver, such as circulating lipids, mediators released by adipose, muscle and liver tissues and pancreatic and gut hormones in relation to diet, exercise and inflammation.

## 1. Introduction

Nonalcoholic fatty liver disease (NAFLD) is associated with a wide pathological spectrum, ranging from indolent liver fat storage, associated with an asymptomatic benign clinical course, to progressive cardiovascular, metabolic and/or liver and kidney diseases with higher cancer risks. Insulin resistance (IR) plays a pivotal role in the pathogenic switch of fatty liver. IR as a hallmark of metabolic syndrome stems from the complex dimensional interplay among inflammation and key circulating mediators, organs and tissues, genetic background and major conditioning factors, such as lifestyle (i.e., diet and physical activity). Here, we review the current knowledge on the dynamics of major circulating effectors/mediators of fatty liver, such as circulating lipids, released compounds from adipose, muscle and liver tissues and pancreatic and gut hormones in relation to lifestyle (i.e., diet and exercise) and inflammation. As renal function is frequently altered in patients with NAFLD, contributing to organ damage progression, the interplay with renal pathophysiology has also been addressed for circulating effectors/mediators other than pancreatic hormones.

## 2. Circulating Lipids

### 2.1. Free Fatty Acids (FFA)

**Figure 1:**
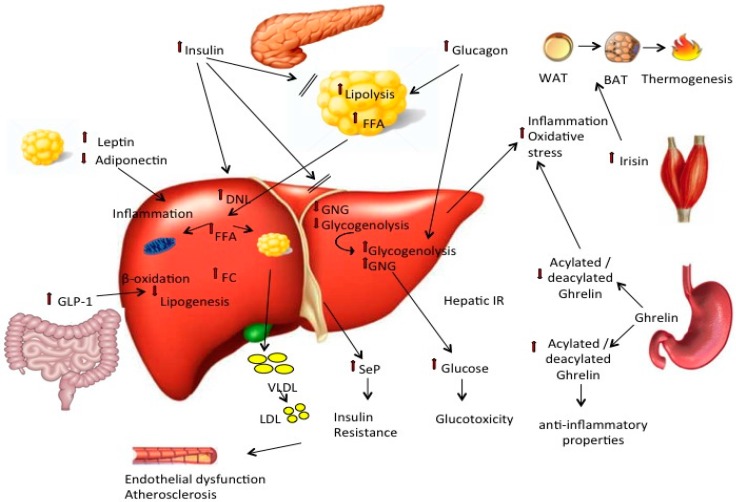
The key metabolic players and the major pathogenic pathways involved in NAFLD. Fatty liver is considered to be the hepatic component of metabolic syndrome. Systemic insulin resistance reduces adiponectin and increases leptin concentrations, while adipose tissue lipolysis is not suppressed (as shown with the “//’’ symbol), despite high circulating insulin levels, and plasma FFA concentration is increased. Increased glucagon levels have also been reported in NAFLD patients. The altered insulin/glucagon ratio promotes DNL, glycogenolysis and gluconeogenesis in the liver, thus increasing hepatic glucose production and hepatic insulin resistance. Several hormones secreted by the gastrointestinal tract regulate glucose/lipid metabolism, as well as food intake and, thus, might be implicated in the development of NAFLD. Impaired GLP-1 secretion and decreased levels of GLP-1 receptors have been reported in the liver of subjects with NAFLD, which further impair hepatic glucose and lipid metabolism. Ghrelin modulates appetite and insulin secretion, and an increased acylated/deacylated ghrelin ratio exerts anti-inflammatory properties.The liver secretes several hepatokines, including SeP, which further enhance insulin resistance, increase the production of small LDL particles that induce atherosclerosis and promote oxidative stress. Adipose tissues secrete adipokine-like leptin and adiponectin that are involved in the modulation of inflammation, fatty acid oxidation and energy expenditure, insulin resistance and insulin secretion. Myokines can also affect glucose and lipid metabolism, e.g., irisin, of which secretion is stimulated by exercise and induces thermogenesis, although its role has not yet been completely elucidated. Small red arrows versus the top: indicate increased concentrations; small red arrows versus the bottom: indicate reduced concentrations.

Circulating FFA, which represent the major source of hepatic fat accumulation in patients with NAFLD, are mainly derived from adipose tissue lipolysis and partly from lipoprotein spill over and are the major fuel substrate for all tissues, except brain during fasting. Thus, their plasma levels are high during fasting and decline after feeding because of the anti-lipolytic action of insulin. In the presence of adipose tissue insulin resistance, FFA levels are high, despite high levels of circulating insulin, because of the resistance to the anti-lipolytic action of this hormone [1,2]. FFAs are involved in the pathogenesis of different metabolic disorders associated with insulin resistance, and different forms of FFA have different implications in cardio-metabolic disorders, ranging from protective to harmful effects [3,4,5,6,7]. Plasma FFAs are reabsorbed in various organs where, if not oxidized, they accumulate under the form of triglycerides within intra-cytoplasmic lipid droplets, and some lipid intermediates, such as or diacyl-glycerols (DAG), promote cell lipotoxicity and mitochondrial dysfunction (Figure 1). Hepatic FFAs can be exported as very low density lipoproteins (VLDL), which can contribute to high circulating triglycerides and low density lipoproteins (LDL), reduced high density lipoproteins (HDL) and an increased risk of atherosclerosis [8].

#### 2.1.1. FFA and Diet

Consistent with the above evidence is that a higher saturated fatty acid (SFA) intake was associated with increased cardiovascular risk [9], whereas a higher intake of polyunsaturated fatty acids (PUFA) showed a protective effect [10], even if contradictory data arose from studies assessing the impact of PUFA supplementation on cardiovascular outcomes [11]. From a practical point of view, a recommended diet should be rich in PUFA and low in SFA.

#### 2.1.2. FFA and Exercise

FFA mobilization and oxidation are higher during low and prolonged versus short and high intensity exercise [12]. In fact, during high intensity exercise, most energy is derived from glucose, while the highest use of FFA as a substrate occurs during low intensity exercise (25% of VO_2_ max).

#### 2.1.3. FFA and Inflammation

Elevated plasma FFA levels, affected also by diet and exercise and resulting from obesity or high-fat feeding, can cause insulin resistance, as well as low-grade inflammation [13]. Recently, the activation of the c-Jun terminal kinase (JNK) pathway by SFA was demonstrated in in vivo investigations [14], contributing to the development of hepatic steatosis and insulin resistance, as well as activation of pro-inflammatory M1 macrophages. Other in vitro studies showed that palmitate may induce endoplasmatic reticulum (ER) and oxidative stress in hepatocytes [15] and trigger the inflammasome via the activation of macrophages through TLR2/1 dimerization [16]. On the contrary, the contribution of unsaturated fatty acids (e.g., oleate, linoleate) to insulin resistance is still debated; they seem unable to affect the cell, but can impact TG storage [17]. Finally, FFAs are the source of diacyl glycerol (DAG), triglycerides and other metabolites, such as ceramides, which are synthesized in the ER of hepatocytes from long-chain SFA, as a substrate [18]. Ceramides were shown to be lipotoxic to pancreatic cells and involved in hepatic insulin resistance [19], but direct evidence of their pro-apoptotic role on hepatocytes is missing [20]. Increased hepatic ceramides and saturated TG and FFA were found in patients with NAFLD [21]. ER stress contributes to NASH progression, and saturated FFAs were shown to induce an ER stress response in hepatocytes and increased levels of ER stress in patients with NAFLD/NASH [22].

#### 2.1.4. FFA and Nonalcoholic Fatty Liver Disease (NAFLD)

The above-mentioned effect of FFA on insulin resistance and low grade inflammation can explain the link between FFA and NAFLD/NASH. Recent in vitro and in vivo studies support the hypothesis that FFAs, which are not esterified and compartmentalized in lipid droplets, may induce irreversible cell damage and trigger pro-inflammatory signaling pathways (lipotoxicity), either alone or in combination with other lipid metabolites [23,24,25]. In addition, other in vitro and in vivo studies have shown that inhibiting hepatic TG synthesis results in an amelioration of hepatic steatosis, but exacerbates liver cell damage due to an increased intra-hepatic accumulation of FFAs [26]. All together, these observations suggest a possible protective role for increased hepatic TG synthesis against FFA-mediated cell toxicity.

#### 2.1.5. FFA and Kidney

The same mechanisms advocating FFA in the pathogenesis of NAFLD/NASH could also be involved in chronic kidney disease (CKD), where the lipotoxicity of FFAs in kidney cells, and in particular on podocytes [27], via ER stress, could explain the pathogenic role of obesity in CKD. Additionally in CKD, although polyunsaturated FAs (such as linoleic acid) probably play a protective role on the kidneys, saturated FFAs, such as palmitic acid, are responsible for intracellular lipotoxicity [28,29].

### 2.2. Cholesterol

Cholesterol is a major lipotoxic molecule, critical in the development of experimental and human metabolic disorders, such as atherosclerosis [30,31]. Different lines of evidence have reported that the accumulation of LDL in vessels make macrophages and smooth muscle cells able to convert esterified cholesterol into cholesterol [31,32,33]. When intra- and extra-cellular accumulation of cholesterol cannot be removed HDL mediated mechanisms, this leads to the generation of cholesterol crystals that, in turn, promote cell death, intima injury and atherosclerotic plaque destabilization [31]. The Seven Countries Study clearly reported a strong link between circulating total cholesterol levels, cardiovascular mortality and diet, with a higher intake of both refined sugar and fat being associated with poor outcomes [31], while dietary fibers have a protective effect [33,34].

#### 2.2.1. Cholesterol and Diet

The influence of cholesterol-free diets on cholesterol serum levels is controversial, in spite of the very large number of different cholesterol-free diet programs [35]. One major reason for this may be the fact that the impact of different diet components on plasma lipid composition is mediated by gut microbiota [36]. The interactions between gut microbiota and dietary lipids in regulating liver and plasma lipid composition, liver gene expression and hepatic cholesterol metabolism were recently shown in germ-free and normally-raised mice [36]. In a study on mice fed lard, gut microbiota increased hepatic, but not serum, levels of cholesterol and cholesteryl esters, while, in mice fed fish oil, neither hepatic nor serum levels of cholesterol and cholesteryl esters were affected [36].

#### 2.2.2. Cholesterol and Exercise

Considering the effect of exercise on cholesterol levels [37], available evidence suggests the particular effectiveness of higher-intensity aerobic exercise [38] and moderate-intensity resistance training [39], with a dose-response relationship between activity levels and increases in HDL cholesterol. However, in order to observe a reduction in plasma cholesterol with exercise training, a reduction in caloric intake and dietary fat during the exercise training program, resulting in a decrease in body weight/body fat, is important [40].

#### 2.2.3. Cholesterol and Inflammation

Cholesterol accumulation in macrophages leads to the induction and secretion of two major inflammatory cytokines, tumor necrosis factor-α (TNF-α) and interleukin-6 (IL-6), which induce inflammation via NLRP3 (nucleotide-binding domain, leucine-rich-containing family, pyrin domain-containing-3) activation and the production of IL1-β and C-reactive protein, suggesting that excess ER cholesterol triggers endogenous cellular events. This proinflammatory activity can explain the link between high cholesterol levels, cholesterol deposition in atherosclerotic plaques and vascular damage [41].

#### 2.2.4. Cholesterol and NAFLD

Lipidomic analyses of NAFLD have demonstrated that, apart from triglycerides, there is also an accumulation of free cholesterol (FC) without a similar increase in cholesterol esters (CE) in both NAFLD and NASH [42] (Figure 1). Again, the above-mentioned cholesterol-related proinflammatory mechanisms, involved in vascular damage, have been also linked to cholesterol-mediated liver damage in NASH [31,32,33]. Along this line, multiple and complex alterations occur in the pathways of cholesterol homeostasis in both NAFLD and NASH [43]. Consistently, statin use has been associated with possible protection from hepatic damage and fibrosis in NAFLD [44].

#### 2.2.5. Cholesterol and Kidney

Several findings suggest the role of systemic and renal lipids in kidney disease development and progression [45]. In fact, lipid loaded cells (i.e., foam cells) are frequently observed in many progressive nephropathies, such as in experimental diabetic kidney disease (DKD) and in focal segmental glomerular sclerosis (FSGS) or minimal change nephropathy in humans [46,47,48]. Secondly, the high prevalence, in African American subjects, of genetic variants of the APOL1 gene, encoding apolipoprotein L1 (a component of HDL), may explain the high susceptibility to nephropathy, in particular to FSGS, of this ethnic group [49]. Finally, some interventions that interfere with lipid accumulation in glomerular cells (podocytes) are effective in reducing kidney damage in some experimental kidney diseases [50]. Although no clear benefit of statin use on chronic kidney disease (CKD) occurrence and/or progression has been demonstrated in a clinical setting [51], the mechanism(s) by which cholesterol might play a causal role in CKD may be more complex than that which is only related to serum cholesterol concentration; again, involving its ability to activate pro-inflammatory mechanisms already involved in atherosclerosis and NASH [45].

## 3. Adipose Tissue Released Compounds

### 3.1. Adiponectin

Adiponectin is a cytokine that is mostly produced by adipocytes, its expression being primarily determined by adipocyte size and insulin sensitivity, with larger, insulin-resistant adipocytes being less productive [52,53]. It is a “protective” adipocytokine, involved in the regulation of glucose and lipid metabolism, as well as in inflammation inhibiting NF-κB and TNF-α production in macrophages; consistent with these data, its serum concentrations are inversely related to obesity and diabetes [54]. Adiponectin levels are inversely related to insulin resistance and are lower in obese subjects and patients with established insulin resistance, e.g., in type 2 diabetes, NAFLD/NASH and hypertension.

#### 3.1.1. Adiponectin and Diet

High-fat, but not low fat, diets were associated with increased adiponectin levels, whereas a modest increase was reported with n-3 polyunsaturated fatty acids (PUFA) supplementation [55]; however, on the contrary, conjugated linoleic acid supplementation showed a reduction in adiponectin levels [55]. In mice, a high-carbohydrate diet was shown to increase adiponectin levels [56], and in humans, Rezvani et al. reported a significant increase of adiponectin levels during the consumption of glucose, but not fructose [57]. Some evidence suggests that adiponectin production by adipocytes is regulated via insulin-stimulated glucose utilization [58].

#### 3.1.2. Adiponectin and Exercise

Mild or moderate physical activity does not change adiponectin levels, though a positive effect was reported with longer exercise [59]. Consistently, increased serum adiponectin levels paralleled the improvement of carotid vascular function in obese individuals undergoing intense exercise and moderate caloric restriction [60].

#### 3.1.3. Adiponectin and Inflammation

While the impact of diet and exercise on adiponectin is controversial, the anti-inflammatory activity of this adipokine, able to inhibit NF-κB and TNF-α production in macrophages, is well established (Figure 1); consistent with these data, its serum concentrations are inversely related to obesity and chronic metabolic disorders, such as insulin resistance and diabetes. In contrast, adiponectin levels are elevated in classic chronic inflammatory/autoimmune diseases unrelated to increased adipose tissue, such as rheumatoid arthritis, systemic lupus erythematosus (SEL), inflammatory bowel disease, type 1 diabetes (T1D) and cystic fibrosis [61].

#### 3.1.4. Adiponectin and NAFLD

Due to the above-mentioned insulin sensitizing and anti-inflammatory activity of adiponectin, its plasma levels are decreased in patients with NAFLD and are associated with fat content [62]. After treatment with thiazolidinediones, adiponectin values increase in NASH as a sign of improvement of hepatic steatosis, necroinflammation and, most importantly, fibrosis [63].

#### 3.1.5. Adiponectin and Kidney

Adiponectin, due to its insulin sensitizing and anti-inflammatory activities, has a protective role on the kidney [64]. In fact, low adiponectin levels were associated with increased albumin urinary excretion and histological evidence of kidney damage, both in experimental and clinical studies [65,66,67]. At odds with these findings, in CKD, the levels of adiponectin are often increased. Whether this finding represents a compensatory phenomenon or just the consequence of the reduced renal clearance of a relatively small molecule (30 kD) and/or of an altered signaling at cellular levels is still a matter of debate [68,69,70].

### 3.2. Leptin

Leptin is a cytokine that is primarily secreted from adipose tissue, with a critical role in the regulation of body weight and fat mass. In obese mice, leptin causes weight loss, increasing energy expenditure and fatty acid oxidation, reducing appetite and triglyceride synthesis and counteracting the lipogenic action of insulin [71]. Its role in humans is less clear-cut; only patients with lipodystrophy have a beneficial effect when treated with leptin, while obese subjects do not lose weight. Circulating leptin is strongly associated with both subcutaneous and visceral fat [72], and different studies have hypothesized that obesity might induce a state of leptin resistance. High leptin levels are associated with reduced insulin secretion, increased gluconeogenesis and reduced glucose uptake, leading to hyperglycemia and ultimately contributing to increased insulin resistance [73,74,75] (Figure 1). Leptin may negatively affect the cardiovascular system by exerting potential atherogenic, thrombotic and angiogenic activities, as well as, even if with contrasting data, leading to cardiac hypertrophy [76].

#### 3.2.1. Leptin and Diet

A higher energy storage is directly related to serum leptin levels [71]. Considering different types of fatty acids: SFAs are associated with increased leptin levels, whereas MUFA and PUFA have an opposite effect [71]. Finally, fiber and higher protein intake increase leptin sensitivity, which induces central satiety [71].

#### 3.2.2. Leptin and Exercise

Available evidence suggests that, while acute and short-term physical activity do not affect leptin levels, longer exercise (at least 60 min) is associated with increased energy expenditure that could lead to leptin decrease [59]. Accordingly, the adiponectin/leptin ratio results as an independent predictor of carotid intima-media thickness (CIMT) alterations [77].

#### 3.2.3. Leptin and Inflammation

Leptin may exert pro-inflammatory activity by the impairment of NO-related vassal relaxation, via increased oxidative stress, and by increased endothelin expression [54,78], by potentiating the effect of angiotensin II, which, in turn, increases leptin synthesis by inducing pro-inflammatory cytokines (e.g., TNF-α, IL-6 and MCP-1 receptor) by increasing the expression of adhesion molecules (e.g., VCAM-1, ICAM-1 and E-selectin). These features could explain why hyperleptinemia is observed in many chronic inflammatory diseases [79,80], such as atherosclerosis, and how it can participate in damage.

#### 3.2.4. Leptin and NAFLD

A recent meta-analysis indicates that circulating leptin levels are higher in patients with NAFLD than in controls, and higher serum leptin levels were associated with an increased severity of NAFLD [81]. This is in agreement with the above-mentioned evidence of inflammatory-mediated damage related to leptin and potential involvement in NASH pathogenesis.

#### 3.2.5. Leptin and Kidney

Leptin is cleared from circulation by glomerular filtration and metabolic degradation in renal tubules, which accounts for the elevated levels of leptin in CKD patients [79]. Given its anoxygenic and pro-inflammatory activities, leptin might contribute to malnutrition and inflammation, often observed in CKD patients, and a consistently higher risk of cardio-vascular morbidity and mortality [80,82,83]. Again, common inflammatory pathways could account for the role of leptin in kidney damage.

## 4. Pancreatic Hormones and NAFLD

### 4.1. Insulin

Insulin is secreted by the pancreas in response to changes in glucose concentrations that occur after a meal or after hormone release, such as catecholamines or glucagon [2]. Insulin tightly regulates glucose metabolism and plasma concentrations, on the one hand, by promoting glucose uptake in skeletal muscle and liver (for glucose oxidation or glycogen storage), in adipose tissue (where glucose is utilized for triglyceride synthesis) and, on the other hand, by suppressing hepatic glucose production. Insulin also acts on lipid metabolism, as it promotes fatty acid re-esterification into triglycerides in adipose tissue and liver, but also inhibits peripheral adipose tissue lipolysis (Figure 1). Thus, the role of insulin in the development of NAFLD is crucial. In the presence of insulin resistance, the pancreas is stimulated to increase insulin secretion to overcome the defect in peripheral glucose uptake and to decrease hepatic glucose production. Since the pancreas releases secreted insulin into the portal vein and the liver clears most of it, the amount of insulin that reaches the liver is much higher than in the periphery. Thus, when hepatic glucose production rates are high in the presence of high insulin values, it is recognized as a sign of hepatic insulin resistance [84]. Insulin mainly acts in suppressing hepatic glycogenolysis, rather than gluconeogenesis; however, until “hepatic autoregulation” is maintained, fasting glucose concentrations remain within normal ranges (Figure 1). When hepatic autoregulation is lost, both components of hepatic glucose production (i.e., glycogenolysis and gluconeogenesis) are increased and assist in the development of fasting hyperglycemia and type 2 diabetes [85]. Finally, different evidence supports a bidirectional link between insulin resistance and chronic inflammation. However, this topic is not the main goal of the present paper, while being the object of a huge debate in the literature, as reported in different reviews [86,87,88].

#### 4.1.1. Insulin and Diet

A carbohydrate-rich diet (with a high glycemic index) determines higher glucose excursion and triggers a higher insulin secretion rate. Moreover, both lipids and amino acids determine increased insulin secretion; oral amino acids elicit a stronger and sustained insulin secretion, as compared to amino acids given intravenously [89]. In addition, lipids have an incretin effect, and a diet high in saturated fats determines insulin resistance and a higher glucose-stimulated insulin secretion (GSIS) [90]. A sustained increase in plasma free fatty acids by long-term intralipid infusion increases GSIS, but this response was found to be impaired in non-diabetic subjects, genetically predisposed to develop type 2 diabetes [91].

#### 4.1.2. Insulin and NAFLD

Insulin promotes de novo lipogenesis (DNL) and glyceroneogenesis [25] (Figure 1). Both pathways are increased in NAFLD, even in non-diabetic patients, contributing to the synthesis of hepatic triglycerides and the promotion of hepatic steatosis [92]. In addition, patients with NAFLD have increased hepatic synthesis of palmitate through DNL, and this increases the risk of lipotoxicity and cell damage [25,93]. Finally, insulin, in the context of insulin resistance, prompts fibrogenesis by stellate cells [94,95]. Most patients with NAFLD have normal fasting glucose concentrations, but high levels of fasting insulin and high hepatic insulin resistance. Thus, it is not surprising that NAFLD is a major risk factor for the development of type 2 diabetes.

#### 4.1.3. Insulin and Exercise

Exercise increases the demand of glucose in the periphery (muscle), and thus, there is a demand for increased endogenous glucose production (EGP). However, since glucose is immediately used by the muscle to produce ATP, glucose concentrations are usually stable, and thus, there is no stimulus for an increase in insulin secretion. However, other hormones, such as glucagon and catecholamine, are increased during exercise and stimulate EGP [12,96].

### 4.2. Glucagon

Glucagon is produced and secreted from alpha cells located in clusters of endocrine cells, in the islets of Langerhans, distributed throughout the pancreas [97]. Glucagon secretion is found to be increased, not only in diabetes, but also in several insulin resistant states, including NAFLD [98]. The role of glucagon is opposite that of insulin (Figure 1); glucagon stimulates glucose production via activation of hepatic glycogenolysis and gluconeogenesis by inhibition of glycolysis [98]. It also regulates fatty acid metabolism via stimulation of peripheral lipolysis, reduction of malonyl-CoA and stimulation of fatty acid oxidation [99]. However, the most recent data indicate that glucagon is also involved in amino acid metabolism, both because amino acids can stimulate glucagon secretion and because glucagon can stimulate protein metabolism [98].

#### 4.2.1. Glucagon and Diet

Glucose is the most important regulator of pancreatic glucagon secretion. In normal glucose tolerant (NGT) subjects, when glucose concentrations are high, glucagon secretion is suppressed, and when there are low glucose concentrations, glucagon secretion is increased, securing an essential supply of energy (i.e., glucose) to the central nervous system and muscles. In patients with diabetes, glucagon concentrations are elevated in the fasting state and fail to decrease appropriately, or even increase, during an oral glucose tolerance test (OGTT) or after ingestion of a mixed meal [100,101,102]. Certain amino acids, such as glutamine, alanine and arginine, are also important glucagon secretors, with the latter being the most potent stimulatory amino acid [103,104]. Fat intake also increases glucagon secretion [105].

#### 4.2.2. Glucagon and NAFLD

Since glucagon stimulates lipolysis and reduces lipogenesis [99], glucagon was proposed as a therapy option for hepatic steatosis [106]. Similarly, it was thought that the reduction of glucagon signaling, i.e., via the use of glucagon receptor antagonists, might lead to the accumulation of lipids in the liver [107]. However, more recent studies [108] have shown that glucagon receptor knockout mice have reduced hepatic lipid contents compared with wild-type mice. The impact of glucagon on NAFLD has not been elucidated. Junker and colleagues [109] have shown that patients with NAFLD have fasting hyperglucagonemia, independent of their glucose tolerance status. According to the authors, this finding suggests that NAFLD might be involved in the generation of hyperglucagonemia in T2D, which is supported by several animal studies [110].

#### 4.2.3. Glucagon and Exercise

Exercise induces an increase in glucagon secretion in order to increase hepatic glucose production and gluconeogenesis. However, although pancreatic hormones are important in the stimulation of EGP during low or moderate intensity exercise, during strenuous exercise (i.e., 80% VO_2_ max), EGP is increased, mainly because of increased catecholamine, while changes in glucagon and insulin are not necessary to stimulate the increase in Ra [96].

#### 4.2.4. Glucagon and Inflammation

Patients with trauma, burns or sepsis normally exhibit increased plasma levels of glucagon, in order to promote gluconeogenesis, increase circulating glucose and compensate for the energetic demand of the body during these extreme situations [111]. Interestingly, significant increases of both glucagon and inflammatory mediators occur after a high fat high carbohydrate meal, as compared with an American Heart Association-recommended meal [112]. Plasma IL-6, a pro-inflammatory cytokine, is elevated in physiological and pathophysiological settings where glucagon is also elevated, such as exercise [113], diabetes [114] and inflammatory stress [115]. Tweedell et al. have demonstrated that IL-6-deficient (IL-6-KO) mice have a blunted glucagon response to acute inflammation compared with their wild-type littermates, while glucagon response is completely rescued by intravenous replacement of IL-6 [116]. Consistent with this, Ortega and colleagues demonstrated that, in patients with altered glucose tolerance, but not in NGT subjects, circulating glucagon levels were associated with inflammatory mediators, such as IL-6 [111].

## 5. Gut Released Hormones

### 5.1. GLP-1

Glucagon-like peptide 1 (GLP-1) is an incretin hormone produced mainly by the L-cells of the gut in response to food intake. GLP-1 has an important role in the regulation of glucose metabolism, since it potentiates insulin secretion and inhibits glucagon release [117,118] (Figure 1). GLP-1 exerts its effect through binding to GLP-1 receptors, which are mainly expressed in the pancreas and brain, but also in the heart, liver, colon and kidney [117]. Other effects of GLP-1 include the central suppression of appetite and the induction of satiety by delaying gastric emptying [117,119]. Other than these classical activities, GLP-1 seems to be able to modulate the function of different key organs by interacting with GLP-1 receptors present in the lung, stomach, liver, colon, kidney and heart. Consistent with these data, growing evidence suggests a direct protective effect of GLP-1 on the cardiovascular system [117,119].

#### 5.1.1. GLP-1 and Diet

GLP-1 release can be stimulated by mixed meals or individual nutrients, including glucose and other sugars, fatty acids, essential amino acids and dietary fiber. Oral, but not intravenous, glucose administration stimulates GLP-1 secretion in humans [120].

#### 5.1.2. GLP-1 and Exercise

Exercise-related studies have shown that healthy people have increased levels of incretin hormones, such as GLP-1, after physical activity [121]. Lee et al. showed higher GLP-1 levels after high intensity vs. low intensity exercise, with matched energy expenditures [122].

#### 5.1.3. GLP-1 and Inflammation

GLP-1 receptor agonists (GLP-1RAS) have anti-inflammatory effects in different cell types, including human umbilical vein endothelial cells, glomerular endothelial cells, monocytes and macrophages [123,124]. GLP-1 levels decreased for a mean duration of 7.5 months in a retrospective analysis of 110 obese patients with T2D who were treated with liraglutide [125]. Consistently, TNF-α induced systemic inflammation and reduced GLP-1 concentrations, thereby reducing the suppression of endogenous glucose production (EGP) during GLP-1 infusion [126].

#### 5.1.4. GLP-1 and NAFLD

In vitro studies have shown that human hepatocytes express the GLP-1 receptor [127,128]. In liver tissue, the expression of GLP-1 receptors is controversial [117], but Svegliati-Baroni was able to demonstrate that, in human livers of subjects with NASH, both the expression and protein content of GLP-1R were decreased compared to subjects without NASH [128]. In subjects with hepatic steatosis, open-label studies have shown that exenatide may improve liver enzymes and decrease steatosis when assessed by magnetic resonance spectroscopy (MRS) [129,130] and even improve histology [131]. A recent study by Armstrong et al. has shown that, after 48 months of double blind treatment with liraglutide vs. placebo (the LEAN study), 39% of patients receiving liraglutide vs. 9% of those receiving placebo had a resolution of definite non-alcoholic steatohepatitis with no worsening in fibrosis [132]. Among the mechanisms that lead to the improvement in liver histology were significant weight loss, reduced FFA flux to the liver, reduced de novo hepatic DNL and the above-mentioned anti-inflammatory activities [133] (Figure 1). All in all, these findings qualify GLP-1RA as a potential candidate for the treatment of NAFLD.

#### 5.1.5. GLP-1 and Kidney

The effects of GLP-1 on glucose metabolism and inflammation, again, can indirectly benefit the kidney. Furthermore, incretin may also have direct renal effects, since its specific receptors have been described both in renal tubular and in glomerular cells [123,134]. One potential mechanism by which GLP-1 may play a nephro-protective role is its natriuretic activity, due to the direct inhibition of two key sodium transporters (Na-hydrogen exchanger-3 and sodium-glucose co-transporter-2) at the tubular level [124]. Furthermore, GLP-1 might also have a positive hemodynamic effect on the kidney by its stimulating and inhibitory effects on atrial natriuretic peptide (ANP) and angiotensin 2, respectively [125].

### 5.2. Ghrelin

Ghrelin is a hormone that is mainly derived from the stomach and duodenum, with a key role in growth hormone release and in food intake control by inducing appetite and controlling energy expenditure [135]. Ghrelin molecules are present as two major endogenous forms, an acylated form, which is the biologically-active form of ghrelin (AG), and a de-acylated form (DeAG) that does not bind to ghrelin receptors [136]. AG is secreted before a meal and disappears more rapidly from plasma than total ghrelin, with an elimination half-life of 9–13 vs. 27–31 min. The main organ that secretes ghrelin is the stomach, where 65%–90% of the circulating ghrelin is synthesized, followed by the small bowel, and in small amounts by other organs, including liver, pancreas, hypothalamus, kidney, liver, fat, muscle and heart (Figure 1). Ghrelin *O*-acyltransferase (GOAT), the enzyme responsible for acylation, was found to be involved in glucose metabolism, insulin resistance, lipid metabolism dysfunction and inflammation [137,138]. GOAT is expressed in several organs, mainly in the gastrointestinal tract, but also in the central nervous system, pancreas, heart, kidney, muscle, tongue, testis, thymus and adipose tissue, but not in the liver.

#### 5.2.1. Ghrelin and Diet

Ghrelin levels (both AG and DeAG) increase with prolonged food deprivation and prior to meal time, while decreases in weight gain, adiposity and in the post-prandial phase with a magnitude proportional to caloric intake and macronutrient content [135,139,140]. GOAT expression and activity and, thus, the availability of AG are modified by dietary lipids, in particular by the availability of short and medium chain fatty acids [138]. Specifically, in a trial using isocaloric beverages, mostly containing fat or carbohydrates or proteins, the lipid drink was the least effective, and the protein drink was the most effective in lowering ghrelin levels, while the carbohydrate drink induced the largest drop in ghrelin levels and was then followed by a significant rebound [141]. Van Name et al. studied the AG response to glucose and fructose beverage in lean and obese adolescents (IS or IR). They found that AG levels were suppressed after either glucose or fructose consumption in lean subjects. In obese IS subjects, AG suppression was higher after glucose as compared to fructose consumption, whereas in obese IR subjects, suppression of AG was blunted following fructose consumption [142]. Thus, it would appear that, in addition to obesity in adolescents, the presence of insulin resistance further limits the capacity of fructose to suppress this key orexigenic hormone and may continue to promote hunger and overconsumption of fructose (or other calories), particularly in obese adolescents who are insulin resistant.

#### 5.2.2. Ghrelin and Exercise

Contradictory results exist on the effect of physical activity on ghrelin levels. Short-term running, cycling or rowing exercise do not alter plasma total ghrelin [143,144,145]. On the other hand, Mackelvie et al. showed that daily exercise for five consecutive days (1-h sessions of aerobic exercise) is associated with an increase in plasma concentrations of AG, independent of the acute effect of exercise and from changes in weight or markers of insulin sensitivity. In addition, the increase in AG was more pronounced in normal weights compared with overweight subjects and was associated with an increase in markers of appetite [146]. However, Shiiya et al. report that plasma AG, but not DeAG levels, are suppressed during acute moderate exercise (cycle exercise for 60 minutes at 50% of VO_2_ max) [146]. From a clinical point of view, this seems more reasonable since exercise increases appetite and exercise is associated with an increase in ghrelin levels (total or acylated form). However, more studies are needed to address the links between different forms of exercise, type, intensity and duration and ghrelin yield.

#### 5.2.3. Ghrelin and Inflammation

Ghrelin, and especially AG, exert anti-inflammatory activity by reducing the production of pro-inflammatory cytokines, such as IL-1, IL-6 and TNF-α, via suppression of NF-κB [137]. The anti-inflammatory properties of ghrelin are consistent with the evidence from murine models that ghrelin prevents diabetes [139] and has a protective cardiovascular effect. These anti-inflammatory properties of ghrelin prompt the ghrelin-GOAT system as a promising new target for the treatment of NASH [137]. AG can improve cardiac function by increasing cardiac output, ameliorating cardiac contractility, acting on cardiac remodeling, reducing pulmonary hypertension, reducing fatal arrhythmia after myocardial infarction and leading to vasodilation [139,147,148,149,150].

#### 5.2.4. Ghrelin and NAFLD

Whether ghrelin levels are altered in NAFLD is still controversial, as Marchesini et al. [151] reported low total ghrelin levels, while Mykhalchyshyn et al. [152] found high serum levels of AG in NAFLD compared to controls. However, the above-mentioned effects of ghrelin on energy and lipid metabolism, IR, inflammation and apoptotic cell death, which are common to both obesity and NAFLD, highly suggest its interplay with NAFLD/NASH pathogenesis [137].

#### 5.2.5. Ghrelin and Kidney

In CKD, increased levels of total ghrelin, but not of AG, are frequently observed, due to the reduced metabolic clearance of the total (mainly DeAG) by failed kidneys. The consequently-reduced AG/DeAG ratio might contribute to inflammatory and malnutrition status, which is typical in many CKD patients [153,154].

## 6. Muscle Released Compounds

### 6.1. Irisin

Irisin is a recently-discovered myokine, encoded by the *FNDC5* gene; it is implicated in the regulation of energy homeostasis and metabolism and the interactions between skeletal muscle and other tissues (Figure 1). Irisin can induce the differentiation of white adipose into brown adipocytes, along with upregulation of uncoupling protein 1 (UCP1) expression and an increase in heat production [155,156]. Accordingly, circulating irisin can increase total energy expenditure, thus reducing obesity and insulin resistance [155,156].

#### 6.1.1. Irisin and Diet

Results of studies on the effect of diet on irisin concentrations are not unanimous. Some studies report that irisin is not affected by food intake [157], while others indicate that irisin levels are positively associated with increasing fruit intake and negatively associated with meat consumption [158]. Finally, an inverse association between irisin and higher caloric intake has been shown [159].

#### 6.1.2. Irisin and Exercise

The reported relationship between irisin and exercise are also contradictory. Some reports have claimed increased irisin serum levels in subjects who exercise [156], while a recent meta-analysis reported that chronic exercise training leads to significantly-decreased circulating irisin levels in randomized controlled trials only, with evidence remaining inconclusive in some other studies [160].

#### 6.1.3. Irisin, NAFLD and Inflammation

To the best of our knowledge, there is only one study in which lower irisin levels were independently associated with higher intrahepatic triglyceride content, as assessed by 1H magnetic resonance spectroscopy [161]. However, in a recent study by Polyzos and colleagues [162], irisin levels were slightly higher in patients with NAFLD and significantly higher in NAFLD patients with portal inflammation, as compared to those without portal inflammation. Contrasting data on higher or lower serum irisin levels in relation with metabolic disorders, diet and exercise are worth further investigation and could be mostly due to the inaccuracy and lack of standardization of commercially-available ELISA assays. Mechanisms underlying the protective metabolic effects of irisin are not well understood and seem to be mostly related to higher induced energy expenditure and not to anti-inflammatory activities, such as NF-κB inactivation [157,158,159].

#### 6.1.4. Irisin and Kidney

CKD patients have been reported to have lower, normal or higher energy expenditures than normal healthy people. The discrepancy among the different studies may be due to many factors related to the type of CKD stage, different therapies and also other, as of yet, unrecognized factors [163,164]. In this complex picture, a recent paper reported an inverse relationship between serum irisin levels and intima-media thickness in dialysis patients [165]. It is also well known that malnourished CKD patients have a worse outcome compared, not only with normally-nourished, but even obese CKD patients. Irisin levels have been found to be lower in CKD patients, and its concentrations were directly dependent on renal function and were related to the components of metabolic syndrome [166,167]. Furthermore, higher irisin levels were associated with sarcopenia in peritoneal dialysis patients [165]. On the basis of these considerations, irisin has been suggested as a candidate for the malnutrition status, often found in the more advanced stages of CKD. However, as for liver diseases, the role and the mechanisms by which irisin affects CKD remain to be further investigated.

## 7. Liver-Released Compounds

### 7.1. Selenoprotein P

Selenoprotein P (SeP; encoded by *SEPP1* in humans) is a secretory protein produced mainly by the liver [168,169] that functions as a selenium transporter from the liver to the rest of the body [170,171]. SeP functions as a hepatokine that contributes to insulin resistance in type 2 diabetes [172] (Figure 1). Importantly, the RNA interference-mediated knockdown of SeP improves insulin resistance and hyperglycemia in a mouse model of type 2 diabetes, suggesting the suppression of SeP production in the liver [173].

#### 7.1.1. Selenoprotein P and Diet

SeP serum levels are directly correlated with the selenium (Se) diet supply (up to 0.1 mg/kg), and Se plays a pivotal role in homeostasis, with its inextricable U-shaped link with health status. Additional selenium intake may benefit people with low levels, whereas it may adversely affect those with adequate-to-high selenium levels. Individuals with serum or plasma selenium concentration of 122 µg/L or higher should not be supplemented with selenium [174].

#### 7.1.2. Selenoprotein P and Exercise

SeP serum levels represent the biologically-active body Se-pool that was shown to slowly decrease during basic training in both trained and untrained individuals [175].

#### 7.1.3. Selenoprotein P and Inflammation

SeP acts as an intracellular antioxidant in phagocytes, modulating inflammatory response via switching macrophage differentiation from M1 to M2 and, of consequence, limiting pathogenicity and oxidative damage [176,177]. On the other hand, SeP serum levels were shown to be lowered by acute-phase inflammatory response [178,179]. A systemic inflammatory response produces cytokines, inhibiting the expression of SEPP1 and reducing selenium levels; pro-inflammatory cytokines, downregulating the SELP promoter in vitro, can, overall, reduce the anti-inflammatory effects of SeP [180]. The interplay between SeP and inflammation may be the link of such a molecule with atherosclerosis, and some controversial epidemiologic data in type 2 diabetes exist [181]. Higher serum levels, inversely related to adiponectin and hepatic SeP concentrations, were reported in patients with type 2 diabetes, with a direct and independent link between SeP and both serum C-reactive protein levels and carotid intima-media thickness, while lower SeP expression was observed in murine adipocytes [173,182,183]. Differences in diabetes-related inflammation and the U-shaped association between SeP and type 2 diabetes risk, mimicking the U-shaped link of Se with health status, might explain some of the apparently contradictory epidemiologic findings. All of these data are worthy of further studies and validation, but indicate the key role of SeP in inflammatory-related cardiovascular alterations.

#### 7.1.4. Selenoprotein P and NAFLD

SeP was found to be increased in NAFLD patients after correction for confounding factors [184]. However, the role of SeP in NAFLD remains to be well elucidated, even if they are able to act, as mentioned earlier, by their ability to modulate inflammatory response and insulin resistance. In addition, different evidence suggests that metformin improves systemic insulin sensitivity through the regulation of SeP production, suggesting a novel potential therapeutic approach to treating type 2 diabetes [185].

#### 7.1.5. Selenoprotein P and Kidney

SeP is the major carrier transporting selenium to target tissues and organs, including kidneys, were it is taken up by mechanisms, which are dependent, by specific receptor-related proteins [186]. According to Reinhardt and colleagues, in patients with CKD, SeP concentrations increase with impaired renal function (even after correction for age and CRP concentrations), whereas SeP concentrations are significantly lower in dialysis patients [187]. The reasons for the discrepant SeP concentrations among the stages of chronic renal failure are not yet completely defined, though the increased inflammatory status in dialysis patients [188] could play an important pathogenic role.

### 7.2. Fetuin-A

Human Fetuin-A/a2-Heremans-Schmid glycoprotein is an abundant 59-kDa serum glycoprotein, produced principally by the liver (thus, it can be classified as a “hepatokine”), and adipose tissue [189]. It works as a natural inhibitor of insulin receptors in the liver and skeletal muscle [190] and also exerts pro-adipogenic effects and suppresses adiponectin release [191]. Deletion of Fetuin-A improves insulin resistance and dyslipidemia and enhances glucose clearance in mice [192], whereas with genetic variants in humans, Fetuin-A has been associated with type 2 diabetes [193] and is linked with insulin action in adipocytes [194]. Serum Fetuin-A levels have been shown to correlate with metabolic syndrome and its main features [191].

#### 7.2.1. Fetuin-A and Diet

In the general population, circulating Fetuin-A was decreased by alcohol intake and milk/dairy product intake, whereas meat and fish had no effect [195]. Resveratrol and curcumin intake may decrease Fetuin-A release [196].

#### 7.2.2. Fetuin-A and Exercise

Short-term exercise training has been shown to reduce Fetuin-A levels, contributing to improvement in hepatic insulin sensitivity, especially in patients with NAFLD [197], although evidence concerning other exercise regimens is still controversial [198].

#### 7.2.3. Fetuin-A and Inflammation

Fetuin-A does not seem to be directly regulated by inflammation, and no correlation was observed between hepatic inflammation and serum levels in patients with NAFLD [199].

#### 7.2.4. Fetuin-A and NAFLD

Increased Fetuin-A has been reported in obese children and lean adults with NAFLD [199,200]. In patients with NAFLD, Fetuin-A levels were associated with the severity of steatosis, were influenced by genetic risk factors for hepatic fat accumulation and also correlated with insulin resistance and metabolic syndrome features [199]. Consistent with the above-mentioned lack of interplay between Fetuin-A and inflammatory response, no correlation was observed between hepatic inflammation and serum Fetuin-A levels in patients with NAFLD [189]. Fetuin-A could affect NAFLD/NASH because it is implicated in the development of insulin resistance and accelerated atherogenesis associated with fatty liver [199,201].

#### 7.2.5. Fetuin-A and Kidney

Fetuin-A is also an inhibitor of vascular calcification, is progressively reduced in patients with renal failure and may modulate the progression of atherosclerosis in patients with chronic kidney disease [202].

## 8. Conclusions

Recent years have brought a great deal of new insights into the complex and dynamic interplay among the multiple effectors/mediators of fatty liver disease. Genomic, meta-genomic and metabolic profiling technologies and other top-down systems biology approaches are well suited for studies of metabolic syndrome and fatty liver disease. The appropriate analysis and interpretation of the physiopathological signatures require a new system of approaches to study and stratify the multifaceted clinical profiles of fatty liver and metabolic syndromes. Bio-statistical modeling will help to identify and combine genomic and meta-genomic determinants of the metabolic pathways and protein interaction networks. Similarly, the systems approach will help to stratify and re-define clinical phenotypes assessing the multiple nature of disease susceptibility and progression. The integration of metabolomic with genomic and meta-genomic markers will improve the understanding of metabolic syndrome and fatty liver disease, and the combined molecular and clinic-pathologic stratification of individuals with metabolic syndrome will allow redefining risks and prognoses, as well as identifying new diagnostic criteria, new markers of disease progression and new endpoints of clinical trials for specific groups of individuals with fatty liver.

## Abbreviations

AG	acylated ghrelin
AMPK	adenosine monophosphate-activated protein kinase
ANP	atrial natriuretic peptide
BAT	brown adipose tissue
BMI	body mass index
CIMT	carotid intima-media thickness
CKD	chronic kidney disease
CRP	C-reactive protein
DAG	diacyl glycerol
DeAG	des-acylated ghrelin
DKD	diabetic kidney disease
DNL	de novo lipogenesis
ELISA	enzyme-linked immunosorbent assay
FC	free cholesterol
FFA	free fatty acid
FNDC5	fibronectin type III domain-containing protein 5
FoxO3a	forkhead box O3a
FSGS	focal segmental glomerular sclerosis
GLP-1	glucagon-like peptide 1
GLP-1R	glucagon-like peptide 1 receptor
GNG	gluconeogenesis
GOAT	ghrelin-ghrelin *O*-acyltransferase
(Oct)-1	organic cation transporter
IR	insulin resistance
IS	insulin sensitive
JNK	c-Jun terminal kinase
LDL	low density lipoprotein
MRS	magnetic resonance spectroscopy
MS	metabolic syndrome
MUFA	monounsaturated fatty acids
NAFLD	non-alcoholic fatty liver disease
NASH	non-alcoholic steatohepatitis
NF-κB	nuclear factor kappa-light-chain-enhancer of activated B cells
NGT	normal glucose tolerance
NLRP3	nucleotide-binding domain, leucine-rich-containing family, pyrin domain-containing-3
NO	nitric oxide
OGTT	oral glucose tolerance test
PUFA	polyunsaturated fatty acids
SC	subcutaneous
Se	selenium
SeP	selenoprotein P
SEPP1	selenoprotein P, plasma 1
SFA	saturated fatty acids
T2D	type 2 diabetes
TLR	toll like receptors
TNF-α	tumor necrosis factor-α
UCP1	uncoupling protein 1
VS	visceral
VLDL	very low density lipoprotein
WAT	white adipose tissue
